# Headache and anxiety/mood disorders: are we trapped in a *cul-de-sac*?

**DOI:** 10.1186/s10194-016-0710-1

**Published:** 2017-01-13

**Authors:** Federica Galli

**Affiliations:** Department of Health Sciences-University of Milan, Via A. di Rudinì, 8, 20147 Milan, Italy

**Keywords:** Migraine, Headache, Anxiety, Depression, Psychiatric comorbidity

After the nth papers on the relationship between migraine and depression [1–3], I think it is the time to open a debate on the meaning of making research on the comorbidity of headache, anxiety and depression. It was 1990, when K. Merikangas published the first pioneering paper on the relationship of migraine, anxiety and depression, outlining the existence of a comorbid association with a bidirectional influence from one disorder to the other(s) and advancing several hypotheses to explain such a comorbidity. Conclusions after 26 years of research on the issue remain the same [1], with the additional complication that comorbid anxiety and depression seems not to be a prerogative of migraine, but of all kind of chronic headache (more frequent and severe are headache attacks more probable the presence of comorbid anxiety and depression-worse is the clinical situation of headache higher the probability of comorbid anxiety and depression). I think the time is mature to admit that we are in a *cul-de-sac*, and we need a way out. If we look to the literature on the issue anxiety/depression and pain other than headache (neck, back, abdominal, musculoskeletal pain, and so on), we will find the same strong comorbid association. Even in rarer clinical disorders (e.g. Burning Mouth Syndrome), we found that anxiety and depression are the most represented comorbid disorders [4]. To complicate the scene, if we look to other common or uncommon, severe or not severe non-painful disorders or diseases (e.g. hearth failure, chronic kidney disease, chronic obstructive pulmonary disease, gastritis and so on) we find again the same strong comorbid association (and I do not open the door on the comorbidity of anxiety and mood disorders with other psychiatric disorders). So, it is the time to advance some consideration on the matter, because the bias of considering anxiety/depression as specifically related to headache (and not strongly related to many different medical conditions as well) constrained us in a no way out. Fruitful lines of research are related to aspects that might help in explaining anxiety and depression components of headache, as personality characteristics, child trauma, abnormal illness behavior, recent life-events, allostatic load (the failure of an organism to achieve stability through change), and so on. New insights could be gained crossing clinical psychological factors with data from imaging studies.

A final warning on the use of the Hospital Anxiety and Depression Scale (HADS) to assess psychiatric comorbidity [2, 3], because we risk misapplication and misinterpretation of findings. The HADS is a useful screening test for detecting symptoms of anxiety and depression *one week* before a probable hospitalization. Symptoms do not mean diagnoses, which need *ad hoc* structured questionnaire and/or clinical interview. Any conclusion based on the HADS is at best speculative, because it does not allow a clear-cut diagnosis of “anxiety” and/or “depression”.

Finally, I suggest the involvement of clinical psychologists and clinical researchers in planning and realizing research on psychological components of headache, because the number of suitable instruments to detect the psychological characteristics of patients is very wide, changes with age and depends on the psychological constructs one chooses to analyse. I do not know if it will drive us out of the sac, but it will unquestionably help!
