# Supplementary material for: Headache and anxiety/mood disorders: are we trapped in a cul-de-sac?
Source: J Headache Pain. 2017 Jan 13;18(1):6. doi: 10.1186/s10194-016-0710-1 (PMC5236036; doi:10.1186/s10194-016-0710-1)
Supplement: Related texts

# Reply: Authors’ response

**Authors:** Timothy J. Steiner, Christian Lampl, Mattias Linde, Christian Wöber, Karin Zebenholzer

**Affiliations:** 2 Norwegian University of Science and Technology (NTNU), Trondheim, Norway; 3 Imperial College London, London, UK; 4 Headache Medical Center, Linz, Austria; 5 Universitätsklinik für Neurologie, Medizinische Universität Wien, Vienna, Austria

Dr Galli has a point. It is both depressing and anxiety-inducing to be in a *cul-de-sac* with no apparent way out. Her suggestion to involve clinical psychologists and clinical researchers in planning and realizing research on psychological components of headache is sensible. Sadly, it does not point to a way out, since she offers no further suggestion on what form that research might take to succeed in this purpose.

Meanwhile, perhaps Dr Galli also misses a point. Regardless of specificity to headache *versus* other pain or chronic disorders (which is not claimed), these associations assessed in the context of population-based studies are highly relevant to needs assessments, formulation of health policy, the structure of health-care provision and resource allocation to it. These studies generally recognise that HADS [5] is not a diagnostic instrument, but its long and respected history of application in epidemiological studies does not support her contention that “Any conclusion based on the HADS is at best speculative”.
